# Effects of Controlled Whole-body Vibration Training on Balance and Fall Outcomes Among Healthy Older Adults: A 6-Week Pilot Study

**DOI:** 10.14283/jarlife.2022.6

**Published:** 2022-07-29

**Authors:** F. Saucedo, E.A. Chavez, H.R. Vanderhoof, V.N. Pradeep Ambati, J.D. Eggleston

**Affiliations:** 1 Department of Kinesiology, Penn State Altoona, Altoona, PA, USA;; 2 Interdisciplinary Health Sciences Doctoral Program, The University of Texas at El Paso, El Paso, TX, USA;; 3 Department of Kinesiology, California State University at San Bernardino, San Bernardino, CA, USA;; 4 Department of Kinesiology, The University of Texas at El Paso, El Paso, TX, USA

**Keywords:** Vibration exercise, fall prevention, balance, retention, walking

## Abstract

**Background:**

Falling is the second leading cause of injury-related death worldwide and is a leading cause of injury among older adults. Whole-body vibration has been used to improve balance and reduce fall risk in older adults. No study has assessed if vibration benefits can be retained over time.

**Objectives:**

The aims of this study were to examine if six-weeks of whole-body vibration could improve balance and fall outcomes, and to assess if benefits associated with the training program could be sustained two months following the final training session.

**Design and Setting:**

Repeated measures randomized controlled design.

**Participants:**

Twenty-four independent living older adults were recruited and were randomly assigned to the whole-body vibration or control group.

**Intervention:**

Participants performed three sessions of whole-body vibration training per week with a vibration frequency of 20 Hz or with only an audio recording of the vibration noise. An assessment of balance and fall outcomes was performed prior to, immediately following, and two-months after the completion of the training program.

**Main Outcome Measures:**

Composite balance scores from the Berg Balance Scale and treadmill fall rates were assessed pre-training, post-training, and two-months post-training.

**Results:**

Seventeen participants completed the study. No between groups differences were found (p<0.05) in the measures of balance or fall rates.

**Conclusions:**

Findings revealed that six weeks of whole-body vibration was not effective in improving balance scores or fall rates.

## Introduction

Falling represents the second leading cause of injury-related death and the leading cause of serious injury in the aging population worldwide (1). An estimated 420,000 individuals die directly from falls or fall-related injuries annually and reports show that falls claim one life every 20 minutes (1). Older adults are more likely to experience severe injuries associated with falling (2), resulting in an annual expenditure of $50 billion for nonfatal falls and approximately $754 billion for fatal falls (2). This presents a significant medical and global economic issue

Older adults experience increased falls risk and the highest fall-related morbidity due to age-related deficits in physical performance, like decreased postural control, sensory loss, and mobility (3). Conventional approaches, such as aerobic, resistance and balance programs have attempted to mitigate these effects (4), but limitations, like access, coordination, and physical ability can limit program adherence and overall program success (5).

Whole-body vibration (WBV) training has been utilized to train and improve functional performance in older adults. Compared to traditional forms of training, WBV can be less strenuous, more portable and cost-effective, and requires minimal exercise experience for operation (4). Participants stand on the oscillating platform and receive low intensity stimulation to the lower extremities, which activates the muscles spindles (6). The motor neurons of the central nervous system are activated resulting in tonic contractions of the lower-limb muscles (6). The stimulation and resulting tonic responses can yield physiological changes and benefits in users (6). Studies have shown that WBV can promote improvements in muscle strength, coordination, and mobility, which play a vital role in reducing falls risk and injury (6-8) . Previous findings from six-week interventions have demonstrated that WBV can lead to increases in neural activation and neuromuscular adaptations, which could yield acute performance benefits and help improve fall outcomes (8).

Despite widespread WBV literature, studies have not directly assessed if WBV can reduce fall rates during walking scenarios, but rather have looked at fall risk factors or fear of falling scores (9). Furthermore, previous studies have not assessed if there is retention of benefits following completion of a WBV program. Only four studies have assessed the lasting effects of WBV and have looked specifically at performance outcomes after a three-week, three-month, and six-month washout period, respectively (9-12) . These studies found that participants were not able to maintain performance benefits following the completion of the interventions (9-12) . Consequently, it is not well-understood if WBV can be effective in reducing fall rates, or if it can yield long-term benefits following completion of a WBV training regimen. Thus, the purposes of this study were to examine if a six-week course of WBV training could improve balance and fall rates in response to simulated slips, and to examine whether any benefits of WBV could be retained over a two-month period after program completion. It was hypothesized that WBV would improve balance and fall rates compared to a control (CON) group. Additionally, it was hypothesized that WBV performance benefits would be apparent in the two-months following the completion of the intervention.

**Figure 1:**
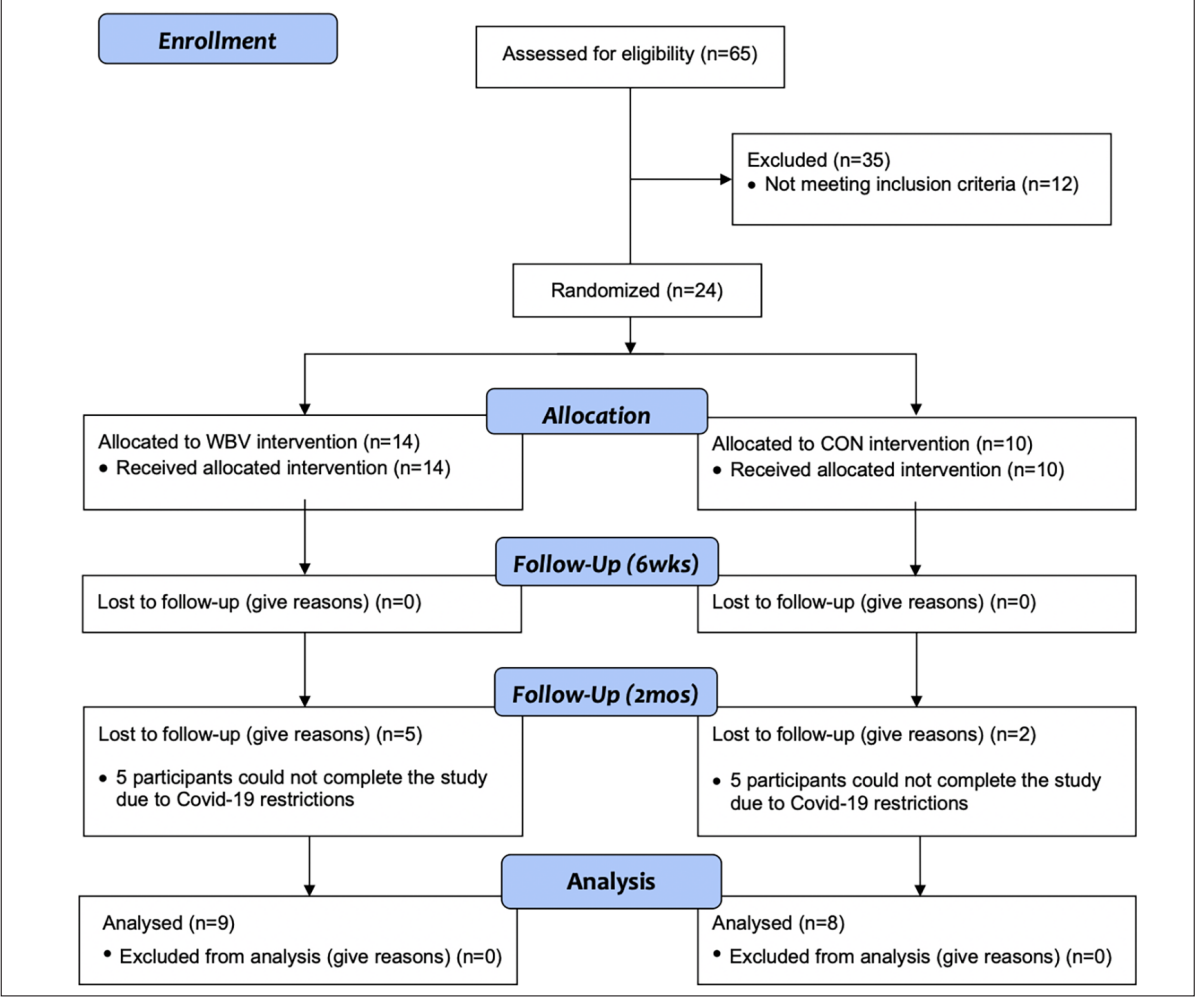
Study flowchart outlining participant recruitment, randomization, and course of study

## Methods

### Participants

An a priori sample estimate of 32 participants (16/ group) was calculated in G-Power 3.1 with a critical alpha-level of 0.05, a large effect size (d= 1.03), and power of 0.80. Twenty-four sedentary older adults with no history of neurological, cognitive, musculoskeletal, cardiovascular, or known gait impairments were recruited. Seventeen participants completed the study (Table 1). Seven participants did not finish due to COVID-19 research restrictions. Participants were randomly assigned into one of two groups (WBV n=9 or CON n=8) using a random number generator and were briefed on all procedures. Participants were blinded to their group assignment, and this information was withheld for the duration of the study. Participants provided written informed consent approved by the University’s Institutional Review Board. This pilot study utilized a randomized controlled design and was performed abiding to the ethical standards described by the 1964 Declaration of Helsinki.

**Table 1:** Group demographic parameters for both CON and WBV participants

Parameter	WBV (n = 9)	CON (n = 8)	p value
Age (years)	71.4+7.1	69.1+5.2	0.730
Sex (female)	7	6	0.563
Body height (m)	1.6+0.1	1.6+0.1	0.355
Body mass (kg)	76.7+13.3	82.9+18.7	0.792
Falls history*	10	5	0.365

### Assessment of fall outcomes

**Figure 2:**
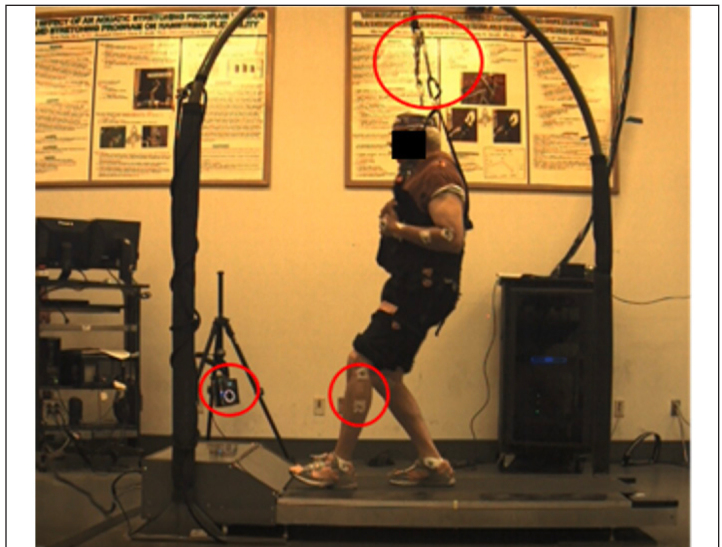
Treadmill, computer, and camera set-up for the slip perturbation protocol. Participants were tethered and secured during all walking trials with a harness and dynamic ropes attached to an overhead arch on the treadmill

Groups were exposed to identical simulated slip conditions on a specialized ActiveStep treadmill (Simbex, Lebanon, NH) (13). While walking, participants donned a full-body harness system (Figure 2) for safety. Participants encountered five unanticipated slip perturbations while walking.

During each session, participants were transferred to the ActiveStep treadmill following a 10-minute warm-up on a regular treadmill (Tracmaster, Newton, KS, USA) where preferred walking speed was determined using established methods (14). Before the simulated slip test, participants were informed that they would experience normal walking first and a “slip-like” movement on the treadmill “later” without knowing when or how the slip would be initiated. Participants were instructed to maintain forward gaze and to try to recover balance and resume walking (if) any slip occurred.

After five trials at preferred walking speed sans slip, participants were then exposed to five slip trials. These trials began with 1.5-second ramp-up, followed by a four-second steady state with a backward-moving belt. After 8-16 (randomized) steps were detected, the top belt accelerated anteriorly, coinciding with the beginning of the next single stance phase. This sudden redirection of the belt induced a forward displacement of the participants’ base of support relative to their center of mass (COM). This slip was unannounced and unpredictable by participants. Lower-body kinematics were captured by a 10-camera motion capture system at 200 Hz (Vicon Motion Systems Ltd., Oxford, United Kingdom) with reflective markers placed on the tenth thoracic vertebrae and sacrum, and bilaterally on the anterior superior iliac spine, iliac crest, posterior superior iliac spine, trochanter, thigh, knee, shank, ankle, heel, and toe. Raw marker trajectory data were exported to Visual3D Biomechanical software suite (C-Motion Inc., Germantown, MD) and were low-pass filtered with fourth-order, zero-lag Butterworth filter (6 Hz) and were used to calculate the body center of mass. The COM-position was computed with respect to trailing heel position during the slip. A COM-position beyond the limits of the trailing heel during the slip phase was classified as a slip. A COM-position located anteriorly with respect to the trailing heel (i.e. within the base of support) during the slip was classified as a recovery. Fall rates were assessed pre-training, post-training, and two-months following the end of the program (Rtn). Fall rates (%) were calculated as the total number of treadmill falls, divided by the total number of treadmill trials for each group.

### Assessment of body balance

Balance was assessed using the Berg Balance Scale (BBS) (15). Participants completed 16 functional balance tasks, each increasing in degree of difficulty. A composite score, out of 56, was calculated to measure performance. Balance was assessed at the three time points described previously.

### Training Intervention

**Figure 3:**
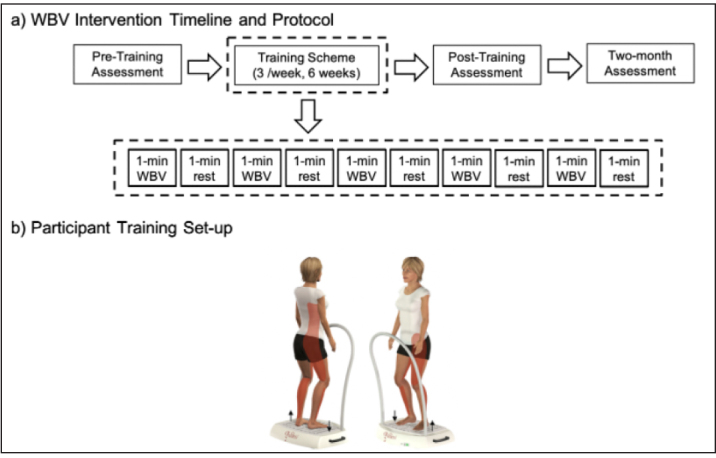
Schematics of (a) the whole-body vibration timeline and protocol breakdown and (b) participant set-up on the side-alternation vibration platform. Vibrations were delivered intermittently at a frequency of 20 Hz and a vibration amplitude of 1.3mm

A side-alternating vibration platform (Galileo Med-L, German) was used (Figure 3). The platform rotated about an anteroposterior axis, so positioning the feet farther from the axis of rotation would result in larger-amplitude vibration. The vibrator stimulated at a fixed frequency of 20 Hz with vibration amplitude set to 1.3 mm, a setting designed to stimulate the stretch reflex and promote muscle function (16). This frequency was selected to maximize comfort and retention in the protocol and to reduce the risk of excess stimulation or resonating of the physiological systems (17). During each training session, participants in the WBV group completed one set of vibration training. The training was intermittent with one-minute vibration sessions followed by a one-minute rest, for a total of 10 minutes. To avoid adverse effects or discomfort while on the vibration platform, knee flexion was maintained at 20º (18). To minimize the shoe-dampening effect, participants stood on the platform barefoot.

The CON group performed an identical protocol, with only an audio recording of the vibrator motor playing during the training to mimic the sound of the WBV protocol (19).

The trainings were repeated three times per week, for six weeks. Successful completion of the programs occurred when each participant completed 18 sessions. At least 24 hours were observed between consecutive training sessions. All sessions were conducted individually under the supervision of the principle investigator to monitor participant status and note any adverse mild effects potentially associated with training sessions (i.e. itching, edema of the legs, soreness) (20).

### Statistical Analyses

Analyses were performed using SPSS software version 24 (IBM, Armonk, New York). A Chi-Square Test was conducted to assess between group differences in baseline characteristics and Fisher’s Exact Test was used to denote significance. Continuous outcomes were compared using an independent t-test. Repeated measures analysis of variance (ANOVA) was used to identify the effect of WBV training on fall rates and balance. The within subject factor was the time instances (pre vs. post vs. rtn) while group (WBV vs. CON) served as the between subject factor. A critical alpha level of p <0.05 was used to determine statistical significance.

## Results

**Figure 4:**
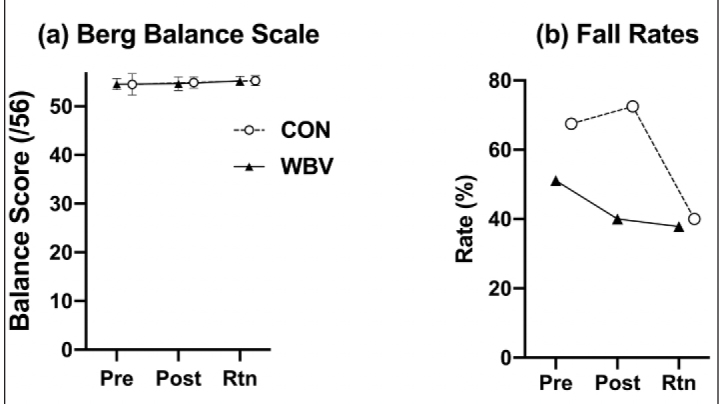
4.a) Group means and standard error bars for (a) Berg Balance Scale composite scores for the pre-test (Pre), post-test (Post), and two-month retention (Rtn) 4.b) Group fall rate percentages for the pre-test, post-test, and two-month retention (Rtn). Fall rates were calculated as the quotient between the total number of falls recorded for the entire group and total number of valid slip trials for the group

Seventeen participants completed all 21 sessions of the study and reported no adverse effects. Baseline characteristics are presented in Table 1. The Chi-Square Test showed no differences in gender between groups (p >0.05) and no differences were identified between groups in age (yrs.), height (m), or mass (kg), or falls history (p >0.05). No significant time by group two-way interaction was detected for the BBS scores or the treadmill fall rates (Figure 4 a-b). The ANOVA revealed no significant main effect of time for the fall rates (p >0.05) or the BBS (p >0.05). Mean and standard deviation values are displayed in Figure 4a-b.

## Discussion

This study examined the effects of a six-week WBV training program on improving balance scores and fall rates. WBV has become an effective alternative to combat age-related deficits to reduce falls risk in older adults, however, studies have primarily focused on the effects of WBV on falls risk factors (4, 6, 21) and have not assessed if the effects of WBV can be effective in reducing actual fall rates. Furthermore, most studies have only examined the acute effects of WBV on balance scores, and treadmill fall outcomes immediately following the commencement of a training session or training program (7, 22, 23). Few studies have actually assessed if any performance benefits exist in the days, weeks, or months, following the completion of a training program (9-12) . Therefore, the aims of this study were to examine to if six-weeks of WBV training could improve balance scores and reduce fall rates in response to simulated slips, and to examine whether WBV benefits could be retained at least two months after completion of the training program. It was hypothesized that participants in the WBV group would improve body balance and fall rates in response to the treadmill slip. Additionally, it was hypothesized that WBV would yield retention effects in both measures after two months.

Results revealed no differences between groups in BBS performance or the fall rates. Participants in both groups experienced no direct benefits throughout the duration of the study associated with the training program. The results did not support our first or second hypothesis. There were no significant between-group differences in performance outcomes observed for the two-month follow-up. These findings indicate that WBV was not able to yield long-term performance benefits.

These findings are aligned with previously reported outcomes, which revealed no performance changes between an eight-week WBV group and an eight-week WBV plus exercise group (24). Although a control group was not used, the findings imply that WBV was no more beneficial than traditional methods. Other findings have also shown that WBV is no more effective than control conditions or traditional methods of intervention (21). These findings contrast those reported previously, which have found that incorporating WBV training into a conventional regimen improved functional performance (23) and balance scores (25). Several other studies have reported improvements associated with WBV, but these too have combined exercise with a WBV regimen (9), (19). One systematic review concluded that WBV can be an integral tool for fall prevention, but the issue remains that training effects in WBV participants may not be immediately apparent when compared to a control group that undergoes conventional exercise or a combination of exercise and WBV (26).

A small proportion of studies have implemented protocols similar to ours with regard to design and vibration intensity (4, 9, 10). The study by Yang, King, Dillon, & Xu reported significant improvements in balance and fear of falling scores after 8-weeks of WBV (4). This study did not include a control group, limiting their ability to claim that improvements in performance were directly associated with the WBV and not confounding factors (4). Positive WBV outcomes may be confounded without a control group; performance improvements can be linked to learning effects, which can occur throughout the duration of the study (4). Despite lacking the control group, the findings from this study as well as the study described above (27) still provide evidence showing the therapeutic benefits of WBV by showing improvements in key fall risk factors, such as cutaneous sensation, fear of falling, and range of motion (4).

Our study did not show that WBV was better than the CON condition. One possible reason for this may relate to the protocol duration. Studies have commonly implemented WBV training periods lasting between three and eight months (21, 28). While the six-week period we chose may have been sufficient to yield neuromuscular adaptations or increase neural activation, which might have aided performance or lead to acute benefits, it is possible that six weeks is not sufficient to obtain training effects. Although, other studies have implemented six-week interventions yielding improved performance in older adults (7, 22).

One possibility is that training intensity and frequency were insufficient to elicit physiological changes linked to the improvements in balance scores and fall rates. The vibration frequency selected for the current study was 20 Hz and it was delivered intermittently for 60-seconds for a total of five-minutes, three times weekly. The 20 Hz frequency was selected to maximize comfort and retention in the protocol and to reduce the risk of excess stimulation or stimulation resembling that of the physiological systems. Vibration frequencies ranging from 12.5 to 20 Hz have been classified as low-intensity, while frequencies from 30-50 Hz have been classified as high-intensity (29). In theory, higher frequencies elicit greater responses from proprioceptors of the lower-extremities, however, studies have shown that WBV interventions utilizing 20 Hz still result in performance improvements (4, 27).

We acknowledge several limitations. The lack of significant findings in our study is potentially attributed to the small sample size. The COVID-19 global pandemic impacted the study sample size, but future studies will aim to increase the sample size to increase study power. Based on the a priori sample size estimation, a total of 32 participants was required to achieve sufficient statistical power. A posteriori power-analysis revealed that with the 17 total participants recruited, the present study yielded a statistical power of 0.22 at the 0.05 level, thereby impacting the likelihood of detecting statistical differences between groups. Given the nature of our protocol, the inclusion criteria narrowed the pool of eligible participants to those that were healthy and high-functioning and considering the small sample size, a ceiling effect could have resulted. For example, scores for the BBS (Figure 4a) showed little variance in performance for both groups. All participants scored close to maximum (56/56), which reduced our ability to discriminate between the two groups. This test may not have been the most suitable for testing balance performance in these participants. Another limitation in the study was the short duration of the WBV intervention. While other studies have shown that six-weeks of WBV can be effective in improving performance (7, 19, 22), no other study has integrated a true control group in their protocol, as we did. Most studies have included an exercise only group for comparison. Therefore, it is possible that six weeks of training is not sufficient to produce benefits in older adults or may only produce acute benefits that were not detected. A thorough assessment of WBV dosage (time and frequency) needs to be conducted to identify an optimal intervention length to produce benefits. Finally, the fall detection method implemented in this study was likely not robust enough to detect a significant reduction in fall rates. Other studies have used more comprehensive approaches to quantify the limits of stability or COM with respect to the base of support (30). Despite modifying these parameters in the present study, the method is justified. If body COM exceeds the posterior limits of the trailing heel during the treadmill slip (e.g. backwards fall), the argument can be made that there is excess instability which could result in a fall. Although there was not a significant change observed between groups in the fall rates (Figure 4b), we speculate that with a larger sample size and the current fall detection method, we can identify a greater reduction in fall rates stemming directly from WBV.

The overall conclusion from this study was that six-weeks of WBV was not effective in improving balance scores or decreasing fall rates among older adults. While the findings from this study did not show statistically significant findings, there are some strengths and clinically significant findings, which merit some attention in future studies. Our study did not look specifically at actual falls, but rather at the outcome of simulated falls or fall rates. This study represents the only one of a few studies to have looked at simulated-slip outcomes and fall rates during treadmill walking in healthy older adults. The results showed a reduction in fall rates in the WBV group compared to the CON group throughout the duration of the study (including retention) (Figure 4b). Although not significant, these findings potentially have important implications for the growing number of aging adults worldwide. Future studies are required to identify the full benefits of WBV in older adults. Studies focusing on performance levels in more frail participants with a history of frequent falls should be top priority as they may unearth more performance benefits associated with WBV.
